# Symptomatic remission affects employment outcomes in schizophrenia patients

**DOI:** 10.1186/s12888-020-02630-z

**Published:** 2020-05-12

**Authors:** San-Ping Wang, Jung-Der Wang, Jer-Hao Chang, Bo-Jian Wu, Tso-Jen Wang, Hsiao-Ju Sun

**Affiliations:** 1 Institute of Allied Health Science, College of Medicine, National Cheng-Kung University, Tainan, Taiwan; 2 Yuli Hospital, Ministry of Health and Welfare, Hualien, Taiwan; 3 Department of Public Health, College of Medicine, National Cheng-Kung University , Tainan, Taiwan; 4 Department of Occupational Medicine, National Cheng Kung University Hospital, Tainan, Taiwan; 5 Department of Occupational Therapy, College of Medicine, National Cheng-Kung University, No. 1, University Road, Tainan, Taiwan; 6 Taoyuan Psychiatric Center, Ministry of Health and Welfare, Taoyuan, Taiwan

**Keywords:** Employment outcomes, Schizophrenia, Symptomatic remission, Taiwan

## Abstract

**Background:**

Remission criteria were proposed by Andreasen et al. for classifying patients with schizophrenia according to the severity of psychopathology. Up to the present time, there have been no cohort studies exploring the association between remission status and employment outcomes in patients with schizophrenia. The study explored whether symptomatic remission is significantly associated with employment outcomes in a two-year longitudinal study.

**Methods:**

All 525 stable patients with schizophrenia in the therapeutic community of a public mental hospital in Taiwan were recruited between 2013 and 2015. Employment outcomes, defined as the cumulative on-the-job duration (months/per year) and income (new Taiwan dollars, NT$/per year), were investigated at the end of 1- and 2-year follow-up periods after enrollment. For repeated measurements, linear mixed models were constructed to examine the association between symptomatic remission and employment outcomes after controlling for potential confounding variables including age, sex, education, type and daily dose of antipsychotics, cognitive function, psychosocial functioning and initial employment type.

**Results:**

The average age of patients was 51.8 years, and 65.3% were males. Among them, 124 patients (23.6%, 124/525) met the remission criteria at baseline. The linear mixed-model analysis showed that patients who had symptomatic remission were employed 0.8 of a month longer (*p* = 0.029) and earned NT$3250 more (*p* = 0.001) within 1 year than those who did not show symptomatic remission.

**Conclusion:**

Our study suggests that assessing symptomatic remission is a useful part of monitoring treatment effectiveness for schizophrenia, and all strategies targeting the bio-psycho-social domains to attain symptomatic remission are paramount to maintaining favorable employment outcomes.

## Background

Schizophrenia is characterized by positive and negative symptoms, such as delusions, hallucinations, disorganized behavior, blunted affect, and reduced motivation [1–3]. In addition, impaired cognitive function is a core feature of schizophrenia [4–7]. The impact of treating schizophrenia on the health care system is a social and economic burden, not only for the caregivers but also for society as a whole [8]. In addition, due to severe symptoms, people diagnosed with schizophrenia can experience frequent relapses and functional deterioration in many domains, such as declined occupational function [9]. Therefore, reducing the severity of symptoms and achieving symptomatic remission is important for treating schizophrenia [10]. From the perspective of providing effective, long-term care and supporting recovery from schizophrenia, the potential utility of consensus criteria for symptomatic remission that delineates a well-defined outcome goal can foster comparisons of effectiveness across treatment modalities and facilitate the development of appropriate rehabilitation programs [10]. Andreasen and colleagues initially defined symptomatic remission in schizophrenia, published by the Remission in Schizophrenia Working Group (RSWG) in 2005, as patients having eight core symptoms of a low severity level over a period of at least 6 months, and as patients with either none or mild symptoms at first episode who seem more likely to develop symptomatic remission [11].

Multidimensional functional outcome measures, such as the Global Assessment of Functioning [12–14], the Personal and Social Performance Scale [15], and the Functional Remission of General Schizophrenia scale [13, 16, 17], have been used in patients with schizophrenia to examine their personal, social, and occupational role functioning [12, 14, 18, 19]. However, few studies have investigated the relationship between symptomatic remission and employment outcomes. According to the bio-psycho-social approach in the International Classification of Functioning, Disability and Health model [20], patients with schizophrenia may exhibit difficulty related to participation in community activities, work activities, daily living activities, and leisure activities. Therefore, it is suggested that researchers focus more on outcomes based on practical disabling experiences and the real-world functioning of patients instead of engaging in excessive reliance on global measures of psychopathology and disability [21].

Indeed, employment is considered a crucial predictor of enhanced functioning for patients with psychotic disorders [19]. Although most people with psychiatric disabilities show a desire to work, they are more likely to experience adverse labor market outcomes due to symptom interference. However, employment contributes substantially to the quality and satisfaction of life is very important to patients with mental disorders [22]. A previous study recruiting 93 patients with first-episode schizophrenia revealed that symptomatic remission was significantly related to a higher employment rate at 1-year and 12-year follow-up sessions [23]. Patients with schizophrenia who are employed consume a lower amount of medication, exhibit lower negative symptom severity and lower excitement symptom severity, and tend to achieve better global functioning [11, 14, 19]. Nevertheless, there were some limitations in prior studies elucidating symptom and employment outcomes, which follow: 1) The multivariate analysis did not control the important baseline confounders associated with employment status, such as type of antipsychotics [14, 24], cognitive function [25], initial employment types [26], psychosocial functioning [22, 27], social support and skills, previous history of successful employment [28], and intrinsic and extrinsic motivation [29]; and 2) There was not a clear definition of employment outcomes. Previous studies defined “full-time students” or “paid workers” as “occupied,” [23] or defined “paid employment” by asking, “Did you work at a job for pay during the past 4 weeks?” [30] instead of using quantitative variables such as wages earned and tenure of job over a period of time [31]. Consequently, the following question related to employment outcomes for patients with schizophrenia is worth exploring: “Does symptomatic remission predict employment outcomes after controlling for confounders among schizophrenia patients after 1 and 2 years?” A study answering this question may illuminate a key factor pertinent to employment outcomes and create more effective strategies in the future for improving work capability and facilitating recovery in patients with schizophrenia.

The aim of this study was to explore the association between symptomatic remission and employment outcomes, including the cumulative duration and income of work, with adjustments for related confounding variables in a two-year longitudinal study.

## Methods

### Rehabilitation model in the therapeutic community

This study was approved by the Ethics Committee of the Institutional Review Board at Yuli Hospital (YLH- IRB-10310) before commencement.

This study was conducted in a psychiatric hospital, Yuli Hospital, Ministry of Health and Welfare, located in eastern Taiwan, with approximately 2000 patients receiving humanistic and patient-centered professional care. The patients in the therapeutic community at the hospital had greater access to occupational rehabilitation and worked outside the hospital when they were stable. The therapists in the community trained the appropriate patients regularly and encouraged their employment in the outside labor market. This multidisciplinary rehabilitation model consisted of identifying patients’ advantaged ability, matching them to appropriate employment, and negotiating with employers for reasonable salaries, especially in supported and sheltered employment. In general, the salary has remained at a fixed rate for a given job, according to predetermined agreements with employers. However, the salary may be adjusted according to a patient’s actual performance, considering efficiency, regularity, and punctuality in the workplace based on a predefined contract among three parties: the patient, the occupational therapists, and the employers. All occupational therapists and employers reached a consensus to assist the workers according to the same standard, that is, embracing impartiality in the acceptance of workers and avoiding undue tolerance of misconduct, unjustified monetary rewards, and unreasonably harsh requirements of workers. If a patient expressed an unwillingness to keep his or her current job, or if there were signs of an unstable psychiatric condition, intervention procedures such as transient cessation of jobs, medication adjustment, and psychiatric counseling were started. After the intervention, if the patient was still unable or unwilling to meet the requirements of the current work, he or she was exempted from current jobs and the possibility of shifting to a sheltered job or workshop was discussed in the team meeting.

#### Participants

All 550 residents present in the therapeutic community of Yuli Hospital from January 2013 to December 2015 were recruited for this study. Inclusion criteria included patients diagnosed with schizophrenia or schizoaffective disorder according to the Diagnostic and Statistical Manual of Mental Disorders, Text Revision, Fourth Edition. Exclusion criteria included those who had an acute psychotic episode that required a transfer to acute psychiatric wards at the stage of enrollment; those who refused to provide consent for evaluation; those with comorbid major chronic diseases, which may have a severe impact on work (e.g., severe physical disability, stroke-related paralysis, bed-ridden); and individuals with significant cognitive deficit (e.g., delirium or dementia). After the content of study procedures, benefits, and risks were fully explained to patients, informed consents were obtained. All patients who signed the informed consent underwent an initial evaluation for 1- to 2-year follow-up assessments regarding related clinical data and employment outcomes. Their initial background data were collected from the sharing dataset with another study, which aimed to validate the Taiwanese Mandarin version [32] of the Personal and Social Performance Scale (PSP) [15].

### Measurements of independent variables and outcomes

Demographic data including age, sex, educational years, age at schizophrenia onset, and types and defined daily doses of antipsychotics on recruitment were collected from the medical records of the patients. The antipsychotics were categorized into typical antipsychotics (TAs), non-clozapine atypical antipsychotics (NCAAs), and clozapine. Patients concurrently using TAs and NCAAs were categorized as NCAAs users. Those who concurrently used clozapine and two other types of antipsychotics were categorized as clozapine users. The antipsychotics were categorized and their dosages were converted to a defined daily dose (DDD) of antipsychotics according to a prior study [33] and from information available on the website of the Collaborating Center for Drug Statistics Methodology of the World Health Organization (http://www.whocc.no/atc_ddd_index/).

#### Positive and negative syndrome scale (PANSS)

The psychopathology of each patient was assessed using the Chinese version [34] of the Positive and Negative Syndrome Scale (PANSS) [35] at baseline. This evaluation was conducted on the basis of an absolute threshold of severity for the following eight core symptoms: delusions (P1), conceptual disorganization (P2), hallucinatory behavior (P3), blunted affect (N1), social withdrawal (N4), lack of spontaneity (N6), mannerisms/posturing (G5), and unusual thought content (G9).

#### Symptomatic remission

Symptomatic remission as defined by the RSWG in 2005 was evaluated by 4 board-certified psychiatrists using the PANSS [10]. The PANSS was assessed only at enrollment. For each patient, medical records and observations provided by medical personnel encompassing the 6 months before enrollment were examined. During the period of 6 months, stable clinical condition was defined as follows: 1) participants did not have a dominant fluctuation of psychiatric symptoms needing adjustment by psychotropic agents or 2) participants did not require transfer to or referral from acute psychiatric wards. Patients were identified to be in a state of symptomatic remission when each one of the scores of the aforementioned eight items of PANSS was less than or equal to 3 and the condition remained stable for at least 6 months.

#### Mini-mental state examination (MMSE)

The Chinese version [36] of the MMSE [37] is a 33-point questionnaire used extensively in clinical and research settings to measure cognitive function. It is 3 points greater than the original version since the Chinese version added 3 questions to increase the discriminant validity in a population with relatively few years of education. Higher scores indicate better cognitive function.

#### Personal and social performance scale (PSP)

Psychosocial functioning was evaluated with the Taiwanese Mandarin version [32] of the PSP [15], specifically developed for patients with severe mental disorders, and includes 4 components: useful social activities, social relationships, self-care, and disturbing behavior. A global PSP score indicates the level of psychosocial functioning; a higher score means better functioning. Higher PSP scores were found to be significantly correlated with better employment status and a useful work role among patients with schizophrenia [22, 27].

#### Rating procedure

All raters, including one certified psychiatric nurse (Y.H.Y.) and three board-certified psychiatrists (C.H.Y., B.J.W., and S.U.) -who had reached a high standard of interrater reliability (intraclass correlations ranged from 0.86–0.95) with the gold-standard raters from the Yuli Hospital research group, rated the PANSS, MMSE, and PSP. All details of the interrater reliability of these ratings were described in prior studies [32, 33].

#### Initial type of employment

The participants received occupational rehabilitation in three types of employment, including 1) the hospital-based workshop (*N* = 323), which provides simple and repeated activities to develop work potential in patients with stable psychiatric symptoms; 2) sheltered employment (*N* = 68), which provides a protective workplace for patients with work potential who cannot work in a competitive workplace, such as a cleaning services (*N* = 37), assistant cook (*N* = 16), car detailer (*N* = 8), or others (*N* = 7); and 3) supported employment (*N* = 134), which has been highly effective for patients to achieve competitive employment in regular employment in an integrated community setting [26], such as house cleaning (*N* = 61), document delivery (*N* = 34), elder care (*N* = 19), guarding (*N* = 12), painting (*N* = 3), and others (*N* = 5).

#### Employment outcomes

Employment outcomes as dependent variables, defined as the cumulative on-the-job duration (months/per year) and income (NT$/per year), were calculated in the first and second year after entry into this study. There were two time points for collection of outcomes: 1) Time 1: 1 year after the entry and 2) Time 2: 2 years after the entry. Dependent variables of Time 1 were cumulative employment duration and incomes from the entry of this study to Time 1. Dependent variables of Time 2 were cumulative employment duration and incomes from Time 1 to Time 2.

### Statistical analysis

Participants were excluded from the regression model analysis if they met the following conditions during the study: 1) those that were proclaimed dead; 2) those that were discharged to their homes; 3) and those that were transformed from remission at baseline to acute relapse, which required transferal to acute psychiatric wards during the study period. For patients with symptomatic remission at baseline, significant differences in variables between those who had acute psychotic relapse and those who did not would be examined.

For repeated measurements within individual patients, the association between symptomatic remission and employment outcomes was investigated using a mixed-effects model analysis [38]. Potential covariates, including age, sex, educational years, type and daily dose of antipsychotics, cognitive function, initial employment type, and PSP were controlled for. All independent variables were assessed once at baseline. To be noted, the main variable of interest in this study, such as remission status, would be adjusted for multiple testing corrections using the Holm Bonferroni method (please see Additional file 1) for avoiding inflation of Type I error [39]. All analyses were conducted using IBM statistics SPSS, version 19.0, and the significance level was set at two-tailed *p* < 0.05.

## Results

At the baseline, 525 residents in the therapeutic community of Yuli Hospital, Ministry of Health and Welfare, between January 2013 and December 2015 participated in this study. A total of 124 patients (23.6%, 124/525) met the remission criteria at baseline. The demographic and baseline clinical data are summarized in Table 1. The majority of the patients were men (65.3%). The average age of the patients was 51.8 years. Except for antipsychotic type, there were significant differences between the remission group and non-remission group in terms of all variables.

**Table 1:** Characteristics at baseline and employment outcomes of subjects (*N* = 525)

		Remission (*n* = 124)	Nonremission (*n* = 401)		
	Total	Mean ± SD (*n*,%)	Mean ± SD (*n*,%)	*T*/Chi-square	*P*-value
Age	51.8 ± 9.84	49.38 ± 9.54	52.55 ± 9.83	3.17**	0.002
Gender (male, %)	343 (65.3%)	70 (56.5%)	273 (68.1%)	5.65*	0.017
Education (years)	9.16 ± 3.62	10.7 ± 3.48	8.67 ± 3.53	−5.40***	0.001
Age of schizophrenia onset (years)	22.49 ± 6.78	24.22 ± 6.80	21.95 ± 6.69	−3.06**	0.002
PANSS	74.32 ± 17.26	57.90 ± 13.4	79.40 ± 15.01	14.29***	0.001
P	14.27 ± 4.12	11.97 ± 3.42	14.99 ± 4.07	7.48***	0.001
N	22.79 ± 6.49	16.19 ± 4.07	24.83 ± 5.69	18.67***	0.001
G	37.26 ± 9.10	29.74 ± 7.12	39.59 ± 8.35	11.86***	0.001
Antipsychotics type				0.17	0.919
FGA (*n*, %)	200 (38.1%)	48 (38.7%)	152 (37.9%)		
NC-SGA (*n*, %)	182 (34.7%)	44 (35.5%)	138 (34.4%)		
Clozapine (*n*, %)	143 (27.2%)	32 (25.8%)	111 (27.7%)		
Defined daily dose of antipsychotics	0.83 ± 0.73	0.72 ± 0.48	0.86 ± 0.79	2.29*	0.023
MMSE	24.98 ± 8.76	29.87 ± 3.88	23.46 ± 9.30	−11.03***	0.001
PSP	53.25 ± 11.30	62.56 ± 7.52	50.36 ± 10.70	−20.04***	0.001
Initial employment type				36.52***	0.001
Hospital-based workshop (*n*, %)	323 (61.5%)	48 (38.7%)	275 (68.6%)		
Sheltered (*n*, %)	68 (13.0%)	23 (18.5%)	45 (11.2%)		
Supported (*n*, %)	134 (25.5%)	53 (42.7%)	81 (20.2%)		
Employment outcomes					
Cumulated duration (months) per year	4.53 ± 5.31	7.21 ± 5.09	3.71 ± 5.11	−9.38***	0.001
Cumulated incomes (NT dollars) per year	5005.3 ± 14,818.5	11,378 ± 26,836.6	3048.6 ± 7091.2	−4.81***	0.001

Note

*PANSS* Positive and negative syndromes scale; *P* positive symptom scale; *N* negative symptom scale; *G* general behavior scale; *FGA* first-generation antipsychotics (typical antipsychotics); *NC-SGAs* second-generation antipsychotics (non-clozapine atypical antipsychotics); Antipsychotic dose: chlorpromazine equivalent; *MMSE* mini-mental state examination; *PSP* personal and social performance

* *p* < 0.05; ** *p* < 0.01; *** *p* < 0.001

After enrollment to the end of this study, no subjects were proclaimed dead or discharged to their homes. During the 2-year follow-up, there were 69 patients (13.1%, 69/525) who presented with acute psychotic relapse and were transferred to acute wards. After treatment and clinical conditions were stabilized, all patients returned to the therapeutic community. The percentage of transformation from those who were categorized into symptomatic remission at baseline to acute relapse afterward was 8.9% (*n* = 11, 11/124). For patients with symptomatic remission at baseline, there were no significant differences in all variables between those who had acute psychotic relapse and those who did not. Finally, the data collected repeatedly on the remaining 514 patients during the 2-year follow-up were analyzed in the regression model.

Table 2 shows the results of the linear mixed model. After controlling for related confounders and adjusting for multiple testing corrections using Holm Bonferroni method, the *p*-value remained significant and unchanged. Symptomatic remission was significantly associated with both employment outcomes (concerning the detailed steps, please see Additional file 1). The patients who had symptomatic remission were employed 0.8 of a month longer (*p* = 0.029) and earned NT$3250 more (*p* = 0.001) than patients who did not show symptomatic remission. The cumulative on-the-job duration was associated with PSP (B = 0.03, *p* = 0.043). Moreover, initial employment types were also found to be significantly associated with employment outcomes. Patients classified with workshop employment had less cumulative work months and incomes than those with supportive employment and those with sheltered employment.

**Table 2:** Linear mixed-effects model for yearly employment outcomes (cumulative work months and incomes) in a 2-year follow-up study (*N* = 514)

Independent variable	Cumulative work months	*P*-value	Cumulative work incomes	*P*-value
	B (S.E.)		B (S.E.)	
Symptomatic remission ^a^	0.80 (0.37)*	0.029 ^a^	3250.4 (1175.8)*	0.001 ^a^
Age	0.00 (0.01)	0.91	−87.3 (46.9)	0.09
Gender	−0.10 (0.28)	0.71	− 132.6 (926.9)	0.95
Male (ref. level)				
Education (yrs.)	0.06 (0.04)	0.18	184.4 (140.8)	0.31
Antipsychotics type		0.43		0.81
FGAs	0.27 (0.34)		343.2 (1129.0)	
NC-SGAs	0.32 (0.33)	0.33	204.8 (1073.5)	0.93
Clozapine (ref. level)				
Defined daily dose of antipsychotics	0.32 (0.24)	0.18	− 762.9 (791.9)	0.56
MMSE	0.01 (0.02)	0.67	13.3 (62.9)	0.85
PSP	0.03 (0.02)*	0.04	9.1 (50.0)	0.87
Time effect				
Time 1 (first yr.)	0.33 (0.15)*	0.02	1537.5 (1765)	0.29
Time 2 (second. yr.) (ref. level)				
Initial employment types				
Hospital-based workshop	−8.44 (0.34)*	0.001	−11,312.9 (1116.2)*	0.001
Sheltered	0.56 (0.44)	0.19	− 6394.8 (1394.2)*	0.001
Supported (ref. level)				

Note

^a^ Adjusted for multiple testing correction using the Holm Bonferroni method, which revealed cumulative work months and cumulative work incomes were all significantly associated with symptomatic remission (the p-value remained significant and unchanged)

^a^ Concerning the steps of conducting Holm Bonferroni method, please see Additional file 1

*FGA* first-generation antipsychotics (typical antipsychotics); *NC-SGAs* non-clozapine atypical antipsychotics; *MMSE* mini-mental state examination; *PSP* personal and social performance

Age, sex, education, antipsychotic types, antipsychotic dose, MMSE and initial employment type were controlled for

* *p* < 0.05; *B* estimated coefficient; *S.E.* standard error

## Discussion

We found that patients recruited in the therapeutic community exhibited an association between symptomatic remission and employment outcomes. This does not necessarily indicate a causal connection. However, we offer the following arguments with which to corroborate such a hypothesis. First, we included only relatively stable patients with symptomatic remission from the therapeutic community. Because they were not comorbid with other major chronic diseases such as stroke and/or physical disabilities or confined to bed because of an aggravated psychiatric condition, the association would not have been confounded by these factors. Second, the significant associations between symptomatic remission and initial employment types and the employment outcomes in our models corroborate previous reports [12, 14, 40–43]. Many investigators have already emphasized that symptomatic remission can be identified as a major factor of functional outcomes in patients with first-episode psychosis [44]. Our results indicate symptomatic remission as a significant predictor of employment outcomes. These findings could be a partial validation of our final models. We not only supported the effects of symptomatic remission on functional outcomes but also extended the definitions of functional outcome by adding to employment outcomes. Third, our participants were followed for 2 years and repeatedly assessed for employment outcomes at the end of each year. Then, we constructed mixed-effects models to control for the demographic factors, types and daily doses of antipsychotics, cognitive function, PSP, and initial employment types. Thus, these determinants cannot confound the results of the final models. Therefore, we tentatively conclude that symptomatic remission is associated with better employment outcomes.

Undoubtedly, employment is not only an essential factor in people’s ability to integrate but is also a stepping stone toward recovery for this population. There are mental health issues linked to unemployment factors, including cognitive impairment, psychotic symptoms, negative symptoms, fear of losing benefits, stigma, and lack of access to employment services [25, 45]. Past studies and meta-analyses have shown that symptomatic remission is usually associated with improved function [14, 19, 46]. A previous study enrolling 2284 schizophrenia patients found non-remitted patients using RSWG criteria were associated with a lack of paid employment, more mental health costs, poorer quality of life, worse global functioning, and more impaired cognition at a 3-year follow-up [30]. However, while many investigators have already emphasized the importance of achieving functional improvement in addition to symptomatic remission [47, 48], our study is the first to quantitatively estimate the employment outcomes for duration and income. Because 124 of the 525 patients showed symptomatic remission in this study, which was higher in proportion to that in other studies [12, 14, 42], we had a good opportunity to observe the employment outcomes.

In our study, MMSE was not included as a significant predictor of vocational outcomes. This does not imply that cognitive function does not predict vocational outcomes. In fact, MMSE is a tool used for screening and follow-up of dementia, for which there are a number of limitations for the clinical practice [49]. The major drawbacks of MMSE is that it exhibits ceiling effects in the detection of mild cognitive abnormalities; that is, in people with higher educational levels, it is possible to have high scores of MMSE even in individuals with mild cognitive impairment or at the very early stages of dementia. MMSE has significant association with a few disorders exhibiting cognitive impairment, such as Alzheimer’s disease, and it is less sensitive to non-cortical cognitive deficits [50]. Cognitive function measured by a comprehensive neuropsychological battery may be more suitable in the current study. There are many neuropsychological batteries such as the MATRICS consensus cognitive battery [51] or the Brief Assessment of Cognition in Schizophrenia [52], as well as other traditional tests for the evaluation of cognition in individuals with schizophrenia. Moreover, since literature indicates that, cognitive impairment is a strong predictor of functional and vocational outcome in schizophrenia [5, 53–55], the lack of an appropriate neurocognitive battery for the assessment of cognitive functioning of patients in this study does not allow any relationship between cognitive functioning and employment outcomes. In short, although MMSE was used as a proxy of cognitive function for controlling the confounding in the current study, it is not an appropriate measure and may create a bias and hinder the reliability of results for similar studies. It is important to use proper tools to assess cognitive function for schizophrenia patients in further studies.

Furthermore, the finding that MMSE was not significantly associated with work outcomes might be accounted for by multicollinearity which may cause distorted model estimation. Cognitive function was found to have influence on psychosocial functioning [4, 6, 33, 56], which was in accordance with our finding that MMSE scores were found to be significantly associated with PSP global scores (r = 0.46, *p* = 0.001). However, a rule of thresholds of correlation coefficients between covariates of |r| 0.7 is an appropriate indicator for multicollinearity [57]. In fact, the greatest correlation coefficient was between MMSE scores and educational years (r = 0.52, *p* = 0.001), which is also less than 0.7. Hence, the model estimation in the current study did not appear to be biased by multicollinearity.

In addition, we found PSP was significantly associated with cumulative duration of work in this study. This finding is compatible with prior studies, showing that a better psychosocial functioning is pertinent to a positive work role and job acquisition for patients with schizophrenia [22, 27]. Our finding indicates that psychosocial functioning is associated with job tenure. For the purpose of achieving favorable employment outcomes, it is important to set up an integrative treatment plan encompassing 4 domains of PSP, such as useful social activities, social relationships, self-care, and disturbing behavior.

### Strength and limitations

Our study is the first to quantitatively estimate the employment outcomes (i.e., duration and income) using mixed-effects model analysis for patients with schizophrenia-spectrum disorders. Nevertheless, this study has the following limitations: 1) Since we recruited only stable psychiatric patients in the therapeutic community of a public mental hospital in Taiwan, and participants were assigned to work settings mainly based on staff members’ perceptions of their needs and capabilities, our results may not be generalizable to all schizophrenia patients, such as those in the community or in other treatment units; 2) Although we tried to control for as many confounders as possible in this study, we were unable to include all vocational predictors [28], such as factors related to the patients’ social support and skills, previous history of successful employment [28], and motivation [29]; and 3) A major limitation of this study is the lack of longitudinal assessment of symptoms and social functioning in patients. Schizophrenia is a disorder with dynamic changes in terms of symptomatic and functional states. For avoiding bias, in our study, those that had acute relapse and were transferred to acute wards were excluded from the regression analysis; those who participated in the current study were all stable patients. Yet, during the 2-year follow-up, remission status and psychosocial functioning were assessed only at baseline. The fluctuation of remission and psychosocial functioning afterward possibly confounded the analysis of employment outcomes. In addition, for some patients, type and dosage of antipsychotics may be changed over a long-term period. Thus far, as we know, in the current setting, participants kept almost a fixed dose and type of antipsychotics, except for those who relapsed and were transferred to acute wards. If possible, a more robust relationship between symptomatic remission and employment outcomes may be confirmed using repeated measurements of remission status and psychosocial functioning as well as type and dosage of antipsychotics.

## Conclusions

In summary, our study reveals that after controlling for potential confounders, employment outcomes in patients with schizophrenia are associated with symptomatic remission. Assessing symptomatic remission would be useful as a part of monitoring treatment effectiveness for schizophrenia, and all strategies targeting the bio-psycho-social domains to attain symptomatic remission are paramount to maintaining favorable employment outcomes. It would be helpful for those who have severe mental illness to return to the community with the promising outcome of regular, long-term employment.

## Supplementary information

**Additional file 1.** Description of Holm-Bonferroni method.

## Data Availability

According to the IRB regulation in Yuli hospital, the data of this study are not available for online access. If the readers needed to gain access to the data, they have to apply for the consent of the IRB and authors with necessary reasons. This is a requirement mandated for this research study by our IRB and funders.

## Abbreviations

RSWG: Remission in schizophrenia working group
PSP: Personal and social performance;
TAs: Typical antipsychotics
NCAAs: Non-clozapine atypical antipsychotics
DDD: Defined daily dose
PANSS: Positive and negative syndrome scale
CMV-PANSS: Chinese version of the positive and negative syndrome scale
P: Positive symptom scale
N: Negative symptom scale
G: General behavior scale
FGA: First-generation antipsychotics
NC-SGAs: Non-clozapine second-generation antipsychotics
Antipsychotic dose: Chlorpromazine equivalent
MMSE: Mini-mental state examination
