# Supplementary material for: Symptomatic remission affects employment outcomes in schizophrenia patients
Source: BMC Psychiatry. 2020 May 12;20:219. doi: 10.1186/s12888-020-02630-z (PMC7216656; doi:10.1186/s12888-020-02630-z)
Supplement: Supplementary file 1 — Additional file 1. Description of Holm-Bonferroni method. [file 12888_2020_2630_MOESM1_ESM.docx]

Appendix 1 Description of [Holm-Bonferroni method](https://en.wikipedia.org/wiki/Holm%E2%80%93Bonferroni_method)

1. **Description**

The steps of a sequential Bonferroni adjustment (i.e, [Holm-Bonferroni method](https://en.wikipedia.org/wiki/Holm%E2%80%93Bonferroni_method)) for multiple testing corrections are as follows:

1. Sort your p-values in ascending order.
2. Refer to them as pi (i.e. p1, p2, p3, etc.)
3. Than you adjust your α-level and compare the p-values against that new α-levels, i.e. you test whether pi≤α/(1+k−i) where k is the number of statistical tests conducted, i.e. the number of p-values calculated.
4. You can stop when pi>α/(1+k−i). Those pi that fall below the sequentially adjusted α-levels are now your significant tests which are adjusted for [multiplicity](https://en.wikipedia.org/wiki/Multiple_comparisons_problem) (after the [Holm-Bonferroni method](https://en.wikipedia.org/wiki/Holm%E2%80%93Bonferroni_method)).
5. **Example**

For example you conducted five tests (α=0.05) resulting in the following p-values:

p1=0.0024, p2=0.0084, p3=0.019, p4=0.027, p5=0.12

The new α-level you compare p1 against is:

0.05/(1+5−1)=0.010.

Since p1≤0.01, you can move on to p2, new α-level you compare p2 against is:

0.05/(1+5−2)=0.0125

Since p2≤0.0125, you can move on to p3, new α-level you compare p3 against is

0.05/(1+5−3)=0.0167

Since p3>0.0167, you can stop.

1. Explanation in the current study (Please see Table 2)
2. Sort p-values about remission (work months and incomes) in ascending order.
3. Refer to them as pi (i.e. p1, p2, p3, etc.)

For p-value of remission (work months and incomes), p1 = 0.001, p2 = 0.029. The number of statistical tests conducted = k =2

The new α-level you compare p1 against is:

0.05/(1+k−i) = 0.05/(1+2−1) = 0.025

Since p1= 0.001≤0.025, you can move on to p2, new α-level you compare p2 against is:

0.05/(1+k−i) = 0.05/(1+2-2) = 0.05

Since p2 = 0.029≤0.05, we can conclude that adjusted p-value for remission (work months and incomes) after the [Holm-Bonferroni method](https://en.wikipedia.org/wiki/Holm%E2%80%93Bonferroni_method) are both significant.

References

1. [Abdi, H. (2010). Holm’s sequential Bonferroni procedure. Encyclopedia of research design, 1.](https://www.utdallas.edu/~herve/abdi-Holm2010-pretty.pdf)
2. [Peres-Neto, P. R. (1999). How many statistical tests are too many? The problem of conducting multiple ecological inferences revisited. Marine Ecology Progress Series, 176, 303-306.](https://www.researchgate.net/profile/Pedro_Peres-Neto/publication/235554723_How_many_statistical_tests_are_too_many_The_problem_of_conducting_multiple_testing_revisited/links/544ff0e00cf201441e9352de.pdf)
